# Vacuolar Sequestration of Paraquat Is Involved in the Resistance Mechanism in *Lolium perenne* L. spp. *multiflorum*

**DOI:** 10.3389/fpls.2017.01485

**Published:** 2017-08-25

**Authors:** Caio A. C. G. Brunharo, Bradley D. Hanson

**Affiliations:** Department of Plant Sciences, University of California, Davis, Davis CA, United States

**Keywords:** chlorophyll fluorescence, dose-response, herbicide absorption, herbicide metabolism, herbicide translocation, polyamines, putrescine

## Abstract

*Lolium perenne* L. spp. *multiflorum* (Lam.) Husnot (LOLMU) is a winter annual weed, common to row crops, orchards and roadsides. Glyphosate-resistant populations of LOLMU are widespread in California. In many situations, growers have switched to paraquat or other postemergence herbicides to manage glyphosate-resistant LOLMU populations. Recently, poor control of LOLMU with paraquat was reported in a prune orchard in California where paraquat has been used several times. We hypothesize that the low efficacy observed is due to the selection of a paraquat-resistant biotype of LOLMU. Greenhouse dose-response experiments conducted with a susceptible (S) and the putative paraquat-resistant biotype (PRHC) confirmed paraquat resistance in PRHC. Herbicide absorption studies indicated that paraquat is absorbed faster in S than PRHC, although the maximum absorption estimates were similar for the two biotypes. Conversely, translocation of ^14^C-paraquat under light-manipulated conditions was restricted to the treated leaf of PRHC, whereas herbicide translocation out of the treated leaf was nearly 20 times greater in S. To determine whether paraquat was active within the plant cells, the photosynthetic performance was assessed after paraquat application using the parameter maximum quantum yield of photosystem II (F_v_/F_m_). Paraquat reaches the chloroplasts of PRHC, since there was a transitory inhibition of photosynthetic activity in PRHC leaves. However, PRHC F_v_/F_m_ recovered to initial levels by 48 h after paraquat treatment. No paraquat metabolites were found, indicating that resistance is not due to paraquat degradation. LOLMU leaf segments were exposed to paraquat following pretreatments with inhibitors of plasma membrane- and tonoplast-localized transporter systems to selectively block paraquat intracellular movement. Subsequent evaluation of membrane integrity indicated that pre-exposure to putrescine resulted in the resistant biotype responding to paraquat similarly to S. These results strongly indicate that vacuolar sequestration is involved in the resistance to paraquat in this population of LOLMU.

## Introduction

*Lolium perenne* L. spp. *multiflorum* (Lam.) Husnot (LOLMU) is a problem weed around the world and causes yield losses in a variety of cropping systems due to its rapid initial development, high biomass production, and plasticity (Hill et al., 1985). Herbicide resistance in LOLMU has been reported in several countries around the world to a variety of modes of action (Heap, 2017). It has an obligate outcrossing, self-incompatible breeding system, which facilitates the dispersal of herbicide resistance traits within and among populations (Loureiro et al., 2016) and, in some cases, results in the accumulation of herbicide resistance traits (Mahmood et al., 2016).

Paraquat (1,1′-dimethyl-4,4′-bipyridinium dichloride) was first discovered in the mid-1950’s, and has been widely used for weed control due to its broad postemergence spectrum of weed control, non-selectivity and soil-inactivity (Hawkes, 2014). Paraquat has a redox potential of -0.466 mV (Homer et al., 1960), acting as a preferential electron acceptor from ferredoxin (Em, -0.430) in the photosystem I complex (PSI). Upon reduction, the paraquat di-cation becomes paraquat mono-cation radical, which in turn transfer an electron to molecular oxygen, producing reactive oxygen species (ROS) (Summers, 1980). Because paraquat returns to its original di-cation state upon electron transfer to ROS, catalytic concentrations of the herbicide in the chloroplasts are sufficient to cause lipid peroxidation and tissue necrosis (Summers, 1980).

Foliar absorption studies have shown that the plant cuticle is not an impediment to paraquat absorption (Bishop et al., 1987). Uptake is generally rapid and maximum absorption often can reach 90% or greater (Soar et al., 2003). Paraquat translocation, conversely, is strongly influenced by light conditions after application. Plants placed immediately under light conditions after paraquat application exhibit restricted paraquat movement. In the dark, however, paraquat is more mobile due to the relatively slower impacts of this light-dependent herbicide on conducting elements and other plant tissues (Preston et al., 2005). Restricted translocation has been recognized as being involved in the mechanism of resistance to paraquat (Yu et al., 2004) as well as to glyphosate (Preston and Wakelin, 2008; Brunharo et al., 2016).

Polyamines are small, polycationic molecules essential to all eukaryotes and, in plants, are associated with growth, responses to stress and other external environmental stimuli, and other crucial physiological processes (Groppa and Benavides, 2008). Cellular uptake of paraquat into plant cells is believed to be primarily mediated by polyamine transport systems (Hart et al., 1992), because of the structural similarity with the natural substrate of the transporters (Fujita and Shinozaki, 2014). More recently, an *Arabidopsis* L-type amino acid (LAT) transporter bound to the plasma membrane and an ATP-binding cassette (ABC) transporter were reported to be involved in paraquat uptake (Fujita et al., 2012; Xi et al., 2012). Once inside the cytoplasm, paraquat has to reach the chloroplasts where its site of action is located, although it is not clear whether paraquat diffuses or is actively transported to the chloroplast stroma (Li et al., 2013). Knockdown of the gene *PAR1*, which encodes a Golgi apparatus localized LAT transporter, reduced paraquat accumulation in chloroplasts, suggesting that LAT transporters are involved, at least partially, in the intracellular trafficking of paraquat (Li et al., 2013).

Polyamines are primarily stored in vacuoles and, because these molecules are involved in several important physiological and biochemical cellular processes, a highly regulated influx/efflux transport system is present in the tonoplast membrane (Kusano and Suzuki, 2015). Transport of paraquat into vacuoles has been suggested to be due to the structural similarities of the herbicide and polyamines, in particular the distance between positively charged nitrogen atoms on both molecules at physiological pH. Non-specific transport of paraquat into and out of the vacuole has been proposed as a mechanism of paraquat resistance in *Lolium rigidum* (Yu et al., 2010).

Because of the widespread occurrence of glyphosate-resistant LOLMU in California (Jasieniuk et al., 2008), many growers use paraquat instead of or in addition to glyphosate in orchards and vineyards for broad spectrum weed control. Recently, poor control of LOLMU with paraquat was reported in a prune (*Prunus domestica*) orchard in California after several paraquat applications (Brunharo and Hanson, 2016), raising the possibility of multiple resistance in this population. The objectives of this research were to confirm paraquat resistance in LOLMU, study the mobility of paraquat under light-manipulated conditions, and evaluate the stability of paraquat and its fate in the plant. The understanding of the mechanism of herbicide resistance in weeds may help elucidate biochemical processes and the fundamental mechanisms by which plants adapt and evolve.

## Materials and Methods

### Source of Plant Material

Seeds from putative multiple resistant (PRHC) LOLMU plants were collected in May 2015 from a prune orchard near Hamilton City (39°45′08″ N, 122°00′58″ W), California, 1 week after a paraquat application was made by the orchard manager. Seeds were germinated in petri dishes after the seed dormancy was overcome by alternating 5°C in darkness with 25°C in light. Seedlings were then transplanted to pots filled with Ron’s Mix soil^1^ and kept in greenhouse until plants reached the BBCH-23 stage (Hess et al., 1997). Plants were treated with 840 g active ingredient (a.i.) ha^-1^ of paraquat to eliminate susceptible individuals from the field-collected seed. Surviving individuals were grown to maturity, bulked and allowed to produce seeds; this generation was also grown to maturity and treated with paraquat. Plants from the resulting F_2_ generation (biotype PRHC) and a previously characterized susceptible LOLMU (S) (Jasieniuk et al., 2008) population from California were used in this research.

### Whole-Plant Dose-Response

PRHC and S seeds were germinated and single plants were transplanted to potting mix as described in the previous section. At BBCH-23 stage, plants were treated with formulated paraquat (240 g L^-1^, Gramoxone SL 2.0, Syngenta Crop Protection, LLC, Greensboro, NC, United States) at rates ranging from 105 to 6720 g a.i. ha^-1^, in addition to a non-treated control treatment, using a spray chamber equipped with an even flat spray nozzle and calibrated to deliver 200 L ha^-1^. A non-ionic surfactant (90% a.i., Activator 90, Loveland Products, Inc, Greeley, CO, United States) was added at a concentration of 0.25% v/v, following manufacturer’s recommendations. Pots were positioned in a completely randomized design with four replications per treatment per biotype and kept in greenhouse with daily maximum temperature of 24°C and minimum of 18°C throughout the experiment. Visual injury was evaluated 7, 14, 21, and 28 days after treatment (DAT) using a scale 0–100%, where 0% represents absence of visual injury and 100% represents complete mortality. At 28 DAT, above ground biomass was collected, dried, and weighed. Log-logistic regression was used to obtain growth reduction by 50% for both biotypes (GR_50_) and the resistance index (RI) (Knezevic et al., 2007). The experiment was repeated and a Levene’s ANOVA test for homoscedasticity of variance was performed before data were pooled across experiments.

### Absorption and Translocation of ^14^C-paraquat

PRHC and S plants were grown under controlled conditions. When they reached the BBCH-13 stage, plants were transplanted to a hydroponic system comprised of 40 mL vials with PTFE/silicon septa filled with a dilute nutrient solution (Moretti and Hanson, 2017). Three days after transplanting, plants were treated with 1.5 kBq of ^14^C-paraquat (specific activity of 32 mCi mmol^-1^, American Radiolabeled Chemicals, Inc, Saint Louis, MO, United States). Radiolabeled herbicide was mixed with a solution containing commercial paraquat (Gramoxone 2.0 SL) and non-ionic surfactant (Triton X-100, 95% purity, Fisher Scientific, Fair Lawn, NJ, United States), to yield a final concentration approximating a spray solution of 105 g a.i. ha^-1^ and 0.25% v/v, respectively. A 1-μL droplet of the solution was placed on the adaxial leaf surface of the youngest fully expanded leaf, 2 cm away from the leaf ligule towards the leaf blade apex, using a blunt-edged syringe (Nandula and Vencill, 2015). Plants were incubated in the dark for 6 h and then treatments were applied under dim light conditions [photosynthetically active radiation (PAR) equal zero]. Paraquat is a fast acting, light-dependent herbicide and, to ensure that the patterns of translocation were maintained after the application of the herbicide, plants were kept in the dark for 16 h after treatment and then were exposed to saturating photosynthetically active radiation (800 μmol m^-2^ s^-1^ PAR) for an additional 14-h period (Preston et al., 2005). Plants were kept in a 24°C growth chamber throughout the experiment and arranged in a completely randomized design. A subset of plants was destructively harvested at 0, 1, 3, 6, 12, and 16 HAT (dark conditions) and at 20, 24, and 30 HAT (light conditions). At each harvest, plants were split into treated leaf, non-treated leaves, and roots; the treated leaves were also rinsed with a leaf-washing solution (Moretti and Hanson, 2017) to quantify non-absorbed ^14^C-paraquat and calculate percentage of absorption. Additionally, 1 mL of solution was collected from each vial to monitor root exudation of paraquat.

Non-absorbed ^14^C-paraquat was quantified with the addition of a scintillation cocktail (Ultima Gold, Perkin Elmer, Walthan, MA, United States) and ^14^C-carbon disintegration measured with a liquid scintillation spectrophotometer (LS 6500, Beckman Coulter, Fullerton, CA, United States). Treated leaves, shoot and roots were oven-dried and then combusted in a sample oxidizer (307 Sample Oxidizer, Perkin Elmer, Waltham, MA, United States) where ^14^CO_2_ was trapped in a specific CO_2_ trapping solution (Carbo-Sorb E, Perkin Elmer, Waltham, MA, United States), mixed with the appropriate scintillation cocktail (Permafluor E+, Perkin Elmer, Waltham, MA, United States), and ^14^C decay quantified with liquid scintillation techniques. Each treatment by harvest combination was replicated four times and the experiment was conducted twice. Data were pooled following the same criteria as the whole-plant dose-response experiment. Absorption of ^14^C-paraquat was calculated as percentage of applied and translocation as percentage of absorbed. Absorption data were subjected to non-linear regressions (Kniss et al., 2011) and translocation data were fit to polynomial models.

### Metabolism of ^14^C-paraquat

PRHC and S were grown, dark- and light-incubated, and treated as described in the absorption and translocation section. In this experiment, 16.6 kBq of ^14^C-paraquat was applied to the youngest fully expanded leaf and plants were harvested at 0, 24, and 48 HAT, where a 32-h light period followed the 16-h dark-incubation period. Liquid nitrogen, a mortar and pestle was used to thoroughly grind whole-plants. Entire samples were transferred to a 50-ml falcon tube prior to the addition of 10 mL of an extraction solution composed of methanol/HCl 0.5 M (6:4). Falcon tubes were sonicated for 30 min at 65°C, centrifuged at 3800 *g* for 45 min, and a 1-mL aliquot was collected from the supernatant phase. To eliminate particulate matter in the 1-mL aliquot, samples were filtered with a 0.45 μm PVDF syringe filter (Millex-HV, EMD Millipore, Tullagreen, Co, Cork, Ireland) prior to being transferred to 2-mL injection vials. An HPLC (1200 Infinity LC, Agilent, Santa Clara, CA, United States) equipped with a mixed-mode column (100 mm × 3 mm ×3 μm, Acclaim Trinity Q1, ThermoFisher Scientific, San Jose, CA, United States) in line with a flow-through radioactivity detector (FlowStar LB 513, Berthold Technologies, Bad Wildbad, Germany) was used to quantify the parent compound and observe any potential metabolites. The mobile phase was composed of 25% ammonium acetate (100 mM, pH = 5, purity > 98%, Sigma–Aldrich, St. Louis, MO, United States) and 75% acetonitrile (99.9% purity, Fisher Scientific, Fair Lawn, NJ, United States), the column oven temperature was set to 30°C and flow rate 0.6 ml min^-1^. The experiment was conducted using a completely randomized design with four replications per biotype at each time point and the experiment was repeated.

### Maximum Quantum Yield of Photosystem II

PRHC and S plants were grown as described in the whole-plant dose-response section. When plants reach the BBCH-23 stage, the youngest fully expanded leaf of each experimental unit was marked and commercial paraquat (Gramoxone 2.0 SL), along with 0.25% v/v non-ionic surfactant (Activator 90), was applied at 105, 420, 840, and 3360 g a.i. ha^-1^. Plants were kept in a growth chamber set at 24°C, 14/10 h day/night, and 800 μmol m^-2^ s^-1^ PAR. To assess the plant photosynthetic performance after exposure to paraquat, the maximum quantum yield of photosystem II (F_v_/F_m_) was measured by dark-adapting the marked leaves with dark-adaption clips (FL-DC, Opti-Sciences, Hudson, NH, United States) for 20 min prior to taking chlorophyll *a* fluorescence measurements with a chlorophyll fluorometer (OS5p+, Opti-Sciences, Hudson, NH, United States) (Maxwell and Johnson, 2000). F_v_/F_m_ measurements were carried out before paraquat application and at 0.5, 1, 2, 5, 24, and 48 HAT and data were expressed as percentage of the initial control values. The experiment was arranged in a completely randomized design with four replications and was repeated. Data were pooled using criteria previously explained.

### Behavior of Paraquat in the Plant Cell

An electrolyte leakage technique (Dayan and Watson, 2011) was adopted with modifications to assess the action of paraquat in PRHC and S after pre-exposure of plant tissue to selective transporter inhibitors. Youngest fully expanded leaves were harvested from PRHC and S plants at BBCH stage 23 by excising whole leaf blades and then sectioning each leaf into 2-cm leaf segments. Leaf segments were rinsed with deionized water to remove electrolytes present on the surfaces and incubated in dark with solutions containing one of four selective transporter inhibitor treatments. Inhibitor treatments included: (1) 100 μM putrescine (98.5% purity, Sigma–Aldrich, St. Louis, MO, United States), (2) 100 μM sodium-orthovanadate (99.8% purity, Sigma–Aldrich, St. Louis, MO, United States), (3) 50 μM verapamil (99% purity, Sigma–Aldrich, St. Louis, MO, United States) and (4) 100 μM potassium nitrate (99% purity, Sigma–Aldrich, St. Louis, MO, United States)]. Solutions also contained 2% sucrose (w/w, 95% purity, Fisher Scientific, Fair Lawn, NJ, United States), 1 mM 2-(*N*-morpholino)ethanesulfonic acid pH 6.5 (MES, Boston Bioproducts, Ashland, MA, United States) and 0.1% Triton X-100.

After 3 h of incubation in the inhibitor solutions, leaf segments were rinsed and transferred to glass scintillation vials containing 5 mL of solution [2% sucrose (w/w) + 1 mM MES] with or without paraquat [25 μM paraquat] and incubated for 14 h. Vials were arranged in a completely randomized design in a growth chamber set at 24°C throughout the experiment. Treatments containing paraquat were used to assess the role of the transporters in the resistance phenotype and treatments without the herbicide were to correct for background effects. After dark incubation, 800 μmol m^-2^ s^-1^ PAR was applied for 12 h to allow paraquat action.

Conductivity measurements were carried out with a conductivity meter and probe (Seven Compact, Mettler Toledo, Columbus, OH, United States and InLab 751-4mm, Mettler Toledo, Columbus, OH, United States, respectively). An initial measurement was taken when leaf segments were transferred to solutions (0 HAT) to use as background conductivity. Measurements were also taken at 11 (dark), 14 (dark), 19 (light), 22 (light) and 26 HAT (light). Each measurement was standardized as a percentage of the maximum conductivity of the sample, obtained by exposing samples to 2000 μmol m^-2^ s^-1^ PAR for 24 h followed by four freeze-thaw cycles (-20°C freezer until solutions froze, followed thawing in a 70°C oven for 30 min). The experiment was repeated and data were pooled using criteria previously stated. Data were analyzed as a 6 by 6 factorial, with treatments as the main factors and incubation time as the subfactor.

## Results

### Whole-Plant Dose-Response

**FIGURE 1:**
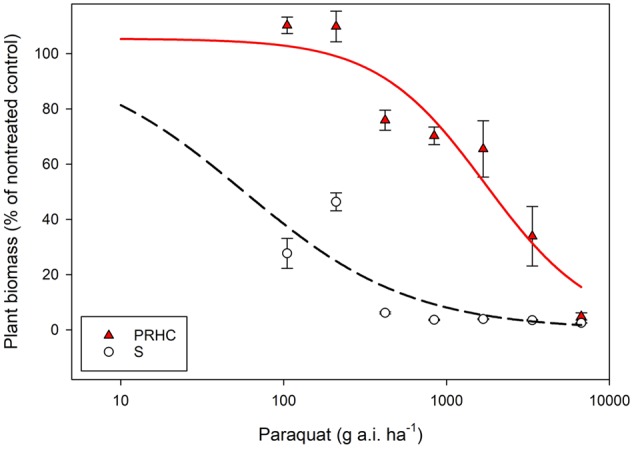
Dose-response analysis of paraquat-resistant (PRHC) and -susceptible (S) *Lolium perenne* L. spp. *multiflorum*. Data points represent plant biomass of PRHC (solid, red triangles) and S (open, black circles) 28 days after paraquat treatment compared to a non-treated control. Bars on data points represent standard errors (*N* = 8). Lines represent three-parameters log-logistic regression of PRHC (red, solid lines) and S (black, dashed lines). *Y = d /1 + exp [b (log x - log e)]*}, where *b* denotes the relative slope around *e, d* is the upper limit, and *e* is the amount of paraquat required to reduce biomass by 50% (in g a.i. ha^-1^).

Paraquat damage was observed in S at all herbicide rates as early as the first visual assessment (7 HAT). Conversely, damage to PRHC leaves was only visible at rates higher than 210 g a.i. ha^-1^ (data not shown). Lower paraquat rates (105 and 210 g a.i. ha^-1^) did not reduce PRHC biomass, whereas these rates reduced S biomass by more than 50% compared to the non-treated control (**Figure 1**). Half of the recommended field rate (240 g a.i. ha^-1^) reduced the biomass of S to near 0%, whereas eight times the recommended rate (6720 g a.i. ha^-1^) was required to induce a comparable response in PRHC. Three-parameter log-logistic regressions were the best fit for the dose-response data. Large standard errors were observed when S data were modeled, particularly the estimated GR_50_, presumably due to the high susceptibility of S to paraquat even at low herbicide rates (**Table 1**). The regression estimate *e* (GR_50_) was 59 g a.i. ha^-1^ for S and 1780 g a.i. ha^-1^ for PRHC, resulting in a 30-fold RI.

**Table 1:** Dose-response analysis of paraquat-resistant (PRHC) and –susceptible (S) *Lolium perenne* L. spp. *multiflorum*.

Log-logistic regression estimates^/a^				
**Biotype^/b^**	***b***	***d***	***e***	**RI^/c^**
PRHC	1.3 ± 0.2	0.6 ± 0.0	1780.4 ± 332.4	30.1 ± 12.7
S	1.6 ± 0.7	0.8 ± 0.0	59.0 ± 22.3	

*^/a^Equation: Y = d /1 + exp [b (log x – log e)]}, where *b* denotes the relative slope around *e, d* is the upper limit, and *e* is the amount of paraquat required to reduce biomass by 50% (in g a.i. ha^*-1*^). ^/b^Values are means ± SE; ^/c^RI = Resistance Index [*e*(PRHC)/*e*(S)].*

### Absorption and Translocation of ^14^C-paraquat

**FIGURE 2:**
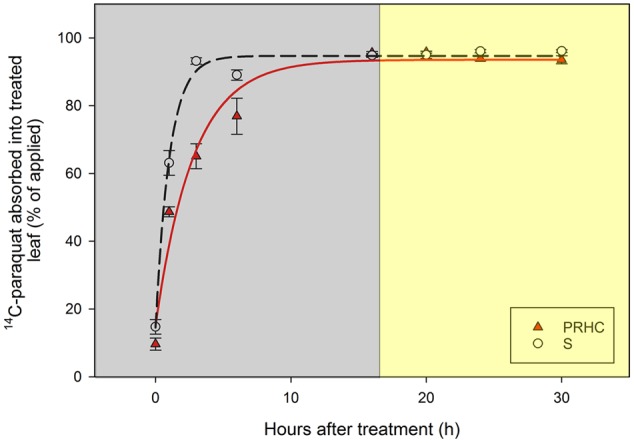
Absorption of paraquat into treated leaf of paraquat-resistant (PRHC, solid, red triangles) and -susceptible (S, open, black circles) *Lolium perenne* L. spp. *multiflorum*. Data points are means and bars represent standard errors. Lines represent asymptotic regressions of PRHC (solid, red line) and S (dashed, black line) after 0, 1, 3, 6, 16, 20, 24, and 30 h after treatment. *Y = (A_max_ x t) / [(10/𝜃) x t_𝜃_ + t]*, where *Y* is the absorption (as percentage of applied), *A_max_* is the maximum percentage of absorption at large values of *t, t* is time, and *𝜃* is an arbitrary percentage of *t*. Dark-shaded area represent timepoints harvested during dark-incubation. Light-shaded area represent timepoints harvested during light-incubation.

Absorption of ^14^C-paraquat into the treated leaf over time reached the upper limit by 16 HAT, within the dark-incubation period, and was best described by rectangular hyperbole models (**Figure 2**). Based on the regression estimate *t_𝜃 = 90_* (time required to 90% of the maximum absorption to be achieved), absorption into S treated leaves was faster (*P* < 0.001) compared to PRHC (**Table 2**). Conversely, *A_max_*, which represents the maximum absorption percentage of the ^14^C-paraquat applied, was similar for both biotypes.

**Table 2:** ^14^C-paraquat absorption regression analysis in paraquat-resistant (PRHC) and -susceptible (S) *Lolium perenne* L. spp. *multiflorum*.

Rectangular hyperbole^/a^ regression estimates^/b^			
	**Biotype**		***P*-value^/c^**
	**PRHC**	**S**	
		
*A_max_*^d^	98.9 ± 1.8	98.5 ± 1.5	>0.05^ns^
*t_90_*^e^	11.4 ± 1.3	4.3 ± 0.6	<0.001

**FIGURE 3:**
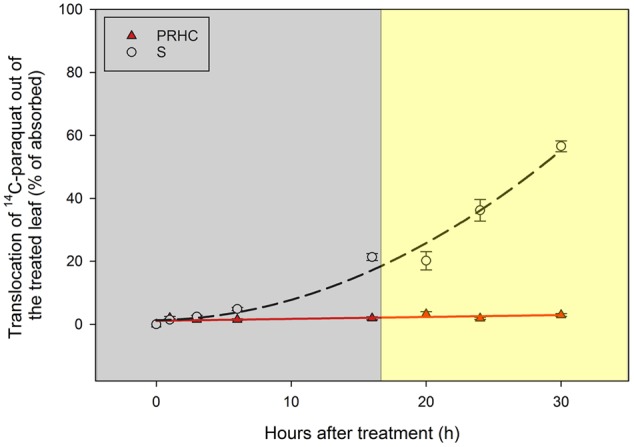
Translocation of ^14^C-paraquat out of treated leaf in paraquat-resistant (PRHC, solid, red triangles) and -susceptible (S, open, black circles) *Lolium perenne* L. spp. *multiflorum*. Data points are means (*N* = 8) and bars represent standard errors. Lines represent polynomial regressions of PRHC (solid red line, *Y = ax + b*) and S (dashed black line, *Y = ax^2^ + bx + c*) after 0, 1, 3, 6, 16, 20, 24, and 30 h after treatment. Dark-shaded area represent timepoints harvested during dark-incubation. Light-shaded area represent timepoints harvested during light-incubation.

Preliminary studies indicated that, when plants are light-incubated after treated with ^14^C-paraquat, the herbicide movement out of the treated leaf is limited in both biotypes (data not shown). For this reason, light conditions before and during the absorption and translocation experiment were manipulated to allow ^14^C-paraquat to translocate before its activity resulted in tissue damage. Translocation of ^14^C-paraquat out of S leaves increased in an exponential fashion (**Figure 3**), reaching 56% by 30 HAT (i.e., 44% remained in treated leaves), whereas movement out of PRHC leaves exhibited a linear response and less than 3% of ^14^C-paraquat was detected in plant parts other than the treated leaves. Paraquat exudation into the hydroponic solution was negligible (data not shown). The methodology adopted to evaluate the absorption and translocation of ^14^C-paraquat yielded total recovery of 97.3 ± 2.9% (sum of radioactivity recovered in all plant parts over total radioactivity applied; data not shown).

### Metabolism of ^14^C-paraquat

The extraction procedure recovered >98% of the applied ^14^C-paraquat. A linear response was obtained with the in-line radioactivity detector (*R*^2^ = 0.99) over the range of ^14^C-paraquat concentrations of 0.8 to 33.3 Bq μL^-1^, with limits of detection lower than 0.8 Bq μL^-1^ (based on signal-to-noise ratio of 3:1 criteria). Elution of ^14^C-paraquat occurred at 2.37 min after sample injection, and no other ^14^C peaks were observed in samples from PRHC and S (data not shown) suggesting a lack of paraquat metabolism.

### Maximum Quantum Yield of Photosystem II (F_v_/F_m_)

**FIGURE 4:**
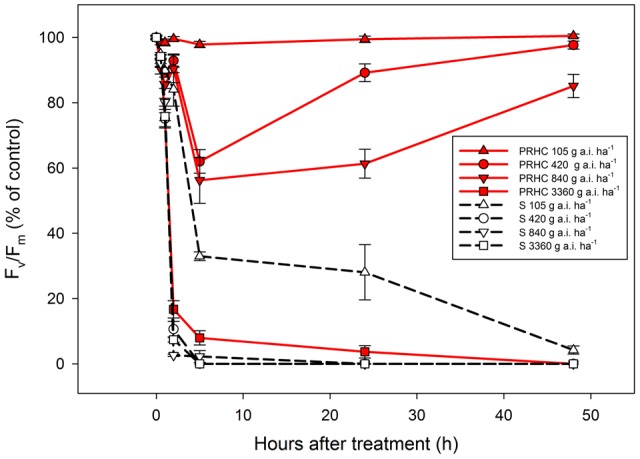
Maximum quantum yield of photosystem II (F_v_/F_m_) after application of paraquat at 105 (triangle up), 420 (circle), 840 (triangle down) and 3360 g a.i. ha^-1^ (square) to paraquat-resistant (PRHC, solid, red lines) and -susceptible (S, dashed, black lines) *Lolium perenne* L. spp. *multiflorum*. Data points are means (*N* = 8) and bars represent standard errors.

Plants exposed to biotic and abiotic stresses exhibit decreases in F_v_/F_m_ values as a consequence of oxidative damage and loss of photosystem II reaction centers. The lowest rate of paraquat (105 g a.i. ha^-1^) did not reduce PRHC F_v_/F_m_, whereas F_v_/F_m_ in S plants was reduced to less than 10% of the non-treated control up to 48 HAT (**Figure 4**). Half (420 g a.i. ha^-1^) and full (840 g a.i. ha^-1^) of the recommended paraquat field rates transiently reduced PRHC F_v_/F_m_ up to 5 HAT, but the photosynthetic performance recovered by 48 HAT, whereas F_v_/F_m_ values in S plants dropped to zero by 48 HAT. The highest rate of paraquat (3360 g a.i. ha^-1^) reduced F_v_/F_m_ in both biotypes to 0% compared to the initial values.

### Behavior of Paraquat in the Plant Cell

**FIGURE 5:**
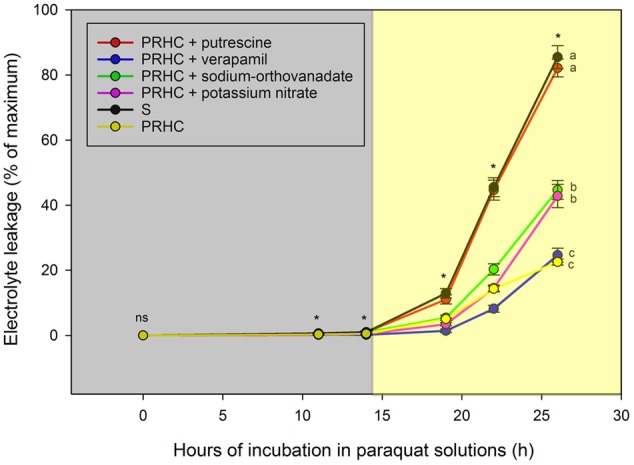
Electrolyte leakage of paraquat-resistant *Lolium perenne* L. spp. *multiflorum* (PRHC) incubated in paraquat solutions for 26 h following pre-treatment with putrescine (red line, red circle), verapamil (blue line, blue circle), sodium-orthovanadate (green line, green circle), potassium nitrate (pink line, pink circle), and no inhibitor (yellow line, yellow circle) and -susceptible with no inhibitor (black line, black circle). Data points are means (*N* = 10), bars represent standard errors, and asterisks means significantly different (*P* < 0.001). Dark-shaded area represent timepoints harvested during dark-incubation. Light-shaded area represent timepoints harvested during light-incubation.

The technique employed to assess the behavior of paraquat in cells of LOLMU pre-treated with inhibitors produced consistent and reproducible results (**Figure 5**). PRHC leaf segments treated with only paraquat exhibited the lowest electrolyte leakage on average; values were statistically similar to leaf segments pre-treated with verapamil. Sodium-orthovanadate and potassium nitrate increased susceptibility of PRHC leaf segments to paraquat in comparison with paraquat-only treatments. Lastly, pre-treatment with putrescine, a polyamine transport inhibitor, followed by paraquat increased electrolyte leakage of PRHC leaf segments to levels similar to S leaf segments treated with paraquat-only, essentially reversing resistance to paraquat.

## Discussion

Whole-plant does-response confirmed paraquat resistance in biotype PRHC based on a RI of 30. These results corroborated grower experience and preliminary research conducted in the prune orchard in Hamilton City, CA, United States. To date, 32 paraquat-resistant species have been reported around the world (Heap, 2017); however, PRHC is the first reported paraquat-resistant LOLMU. Studies with model plants suggests that resistance to paraquat may be caused by mutations that reduce paraquat uptake (Fujita et al., 2012) and/or enhanced stress tolerance by means of increased expression of enzymes that protect the cell against reactive oxygen species (Murgia et al., 2004; Chen et al., 2009). However, these mechanisms confer only marginal tolerance to paraquat (RI < 4-fold) compared to field-selected weed biotypes (Hawkes, 2014) that exhibit RI as high as 352-fold (Moretti et al., 2016).

In tree and vine crops in California, recommended paraquat rates ranges from 700–1120 g a.i. ha^-1^; these rates would be insufficient for full control of PRHC. The obligate-outcrossing self-incompatible nature of LOLMU facilitates the dispersal of herbicide resistance genes within and among populations (Loureiro et al., 2016), and the poor control of PRHC with paraquat allows the spread of paraquat resistance genes to areas where -resistant populations are absent.

The slower ^14^C-paraquat absorption in PRHC compared to S suggests that differential absorption is not a primary cause of resistance in this biotype. Conversely, restricted ^14^C-paraquat mobility seems to be involved in the mechanism of resistance to paraquat, considering that virtually all the herbicide remained in the PRHC treated leaf while more than 50% translocated to other tissues in S plants (**Figure 3**). Non-target-site mechanisms of resistance are extensively reported in the literature, and particular attention to these types of mechanisms is given when paraquat-resistant biotypes are studied (Hawkes, 2014). The facts that paraquat was absorbed, remained in the treated leaf, and symptoms were not observed on treated leaves in PRHC suggests that paraquat is either excluded from the cytoplasm (i.e., away from its site of action) or is absorbed but maintained away from the chloroplasts. In fact, paraquat exclusion to the apoplast has been suggested to be the mechanism of resistance in *Hordeum glaucum* (Preston et al., 1992), whereas sequestration into the vacuole has been proposed to confer resistance in several other paraquat-resistant weed species (DiTomaso et al., 1993; Lasat et al., 1997; Yu et al., 2010).

Paraquat absorption through the plant cuticle does not seem to be light-dependent, since *t_90_* for S and PRHC are reached within the dark-incubation period. Similar conclusions could be drawn about the translocation out of the treated leaf in S. However, basipetal paraquat movement is primarily due to reverse xylem flow driven by the disruption in water relations caused by paraquat damage to leaf tissue (Smith and Sagar, 1966), damage that did not occur in S during the 16 h dark-incubation period. Symplastic movement of ^14^C-paraquat might explain, to a certain extent, the observed translocation of the herbicide out of the undamaged treated leaf during the dark-incubation period, if it is considered that the youngest fully expanded leaf received the treatments; these tissues are generally characterized as source organs. This hypothesis is supported by the fact that polyamines (putrescine and spermidine) are translocated in plants by long-distance transport systems (Friedman et al., 1986). A 40% increase in ^14^C translocation out of the treated leaf was observed in S from the end of the dark-incubation to the end of the light-incubation period.

Paraquat degradation may be driven by biological and physical processes. The former involves an initial demethylation step, followed by ring cleavage of one of the heterocyclic ring (Funderburk and Bozarth, 1967), whereas the latter is given by the formation of 1-methyl-4-carboxypyridinium ion, followed by the formation of methylamine hydrochloride (Slade, 1965). With the observation that paraquat movement was restricted in PRHC, it was hypothesized that, if paraquat transport in plants relies on polyamine transport systems, then paraquat metabolites would no longer be recognized by the transporters, restricting the radiolabeled compounds to the treated leaf. This hypothesis was not supported, however, since paraquat metabolites were not detected in PRHC or S at any timepoint up to 48 HAT. This result is not unexpected because metabolism of paraquat in plants has not been previously reported (Hawkes, 2014), although soil microorganisms may be able to metabolize this quaternary ammonium compound (Funderburk and Bozarth, 1967).

The measurement of F_v_/F_m_ from intact LOLMU leaves indicates that there is a dose-dependent mechanism of resistance acting in PRHC because the highest paraquat dose decreased F_v_/F_m_ to near zero, whereas lower rates did not elicit a comparable response. Similar mechanisms are absent in S, since all doses used led to an irreversible drop in F_v_/F_m_ early in the course of the experiment. Intermediate doses of paraquat (210 and 420 g a.i. ha^-1^) transiently reduced F_v_/F_m_, in PRHC but the photosynthetic apparatus recovered by 48 HAT, suggesting not only that paraquat reaches PRHC chloroplasts, but also that the mechanism of resistance to paraquat does not involve herbicide exclusion from the plant cell as suggested for *Hordeum leporinum* (Preston et al., 2005). However, it seems that the mechanism of resistance to paraquat may be rate-limited to a certain extent because of the dose-dependent response in the resistant biotype. Similar transient, dose-dependent paraquat action was also observed in paraquat-resistant *Conyza canadensis* with more pronounced F_v_/F_m_ recovery when plants were exposed to 500 PAR compared to lower light intensities (Varadi et al., 2000).

Studies with the sub-cellular compartmentation of paraquat in paraquat-susceptible *Zea mays* roots revealed that paraquat is slowly sequestered via a diamine carrier system, whereas the rate of paraquat efflux from the vacuole to the cytoplasm is saturable (Hart et al., 1992; DiTomaso et al., 1993). If it is assumed that LOLMU has an analogous paraquat vacuolar loading systems as *Z. mays*, then two mechanisms of resistance may be supported by our results. Because of the linear rate of paraquat loading into the vacuole (as observed in *Z. mays*), the time in which paraquat is in the cytoplasm is similar in PRHC and S, potentially with a paraquat exclusion mechanism in the chloroplasts preventing paraquat from reaching its site of action. This chloroplast exclusion mechanism might not be sufficiently expressed to eliminate damage from high paraquat doses but may be sufficient at lower doses, explaining the results obtained with the maximum quantum yield of PSII measurements. Reduction of paraquat accumulation in *Arabidopsis* chloroplasts with the gene *PAR1*, which encodes a Golgi-localized LAT transporter, has been shown to confer tolerance to the herbicide (Li et al., 2013). However, the authors also showed that inhibition of the vesicle trafficking only partially alleviated paraquat damage, suggesting that an unknown transporter (possibly a polyamine transporter) is involved in the transport of paraquat to the chloroplast. Another reasonable explanation to the results obtained with the F_v_/F_m_ measurements might be the overproduction of the diamine carrier system that performs paraquat vacuolar loading, enhancing vacuolar sequestration of paraquat while (but not necessarily) maintaining the linear fashion of the natural vacuolar loading observed in *Z. mays* (DiTomaso et al., 1993).

Verapamil, which blocks Ca^2+^ channels (Huang et al., 1994) and inhibits multidrug ABC transporters (Kang et al., 2010) did not increase susceptibility of PRHC to paraquat compared to the paraquat-only control treatment, suggesting that Ca^2+^ channels and multidrug ABC transporters are not involved in the resistance to paraquat in this biotype. The inhibitor of plasma-membrane, tonoplast ATPases and plasma-membrane ABC transporters sodium-orthovanadate (Cocucci et al., 1980; Demichelis and Spanswick, 1986; Finbow and Harrison, 1997), as well as the tonoplast H^+^-ATPase pumps inhibitor potassium nitrate (Sze, 1984), led to intermediate paraquat damage to PRHC leaf segments, suggesting that the mechanism of resistance to paraquat requires energy supplied by the proton gradient across membranes, most likely across the tonoplast membrane. It may be pointed out that sodium-orthovanadate also inhibits plasma membrane ABC transporters (P-type ABC transporters), but since verapamil (all ABC transporters inhibitor) did not provide increased susceptibility of PRHC to paraquat, the involvement of P-type ABC transporters may be unlikely (Shitan et al., 2003).

The observation that pre-exposure of PRHC leaf segments to putrescine reverses resistance to paraquat strongly suggests that polyamine carrier systems are involved in the mechanism of resistance to paraquat in PRHC. Exposure of *Z. mays* roots to putrescine 20 min prior to paraquat incubation has been shown to inhibit polyamine transporter-mediated paraquat transport by up to 65% (Hart et al., 1992).

Polyamine transport can be accomplished by carriers bound to the plasma membrane (Hart et al., 1992) and tonoplast (Pistocchi et al., 1988). Because it was observed in this research that paraquat reaches PRHC chloroplasts, it may be inferred that plasma membrane-bound polyamine carriers do not have a major role in the mechanism of resistance in PRHC, eliminating the possibility of paraquat exclusion to the apoplast being involved in the resistance mechanism.

## Conclusion

Poor weed management practices, particularly overreliance on a single/few herbicide modes of action, have frequently been associated with the selection of herbicide-resistant weed biotypes around the world. Paraquat used to control glyphosate-resistant LOLMU has selected for a multiple resistant biotype in a prune orchard in California; this biotype withstands paraquat at up to three times the maximum field rate that tree and vine growers are allowed to use in this region. The restricted translocation of ^14^C-paraquat in PRHC observed seems to be primarily caused by the vacuolar sequestration of the herbicide mediated by tonoplast-bound polyamines transporters sensitive to the inhibitor putrescine. These findings are supported by the fact that PRHC photosynthetic apparatus is sensitive to paraquat and that paraquat is stable in the plants.

## Author Contributions

CB and BH conceived and designed the experiments. CB conducted the experiments and analyzed the data. CB and BH wrote the paper. All authors have read and approved this manuscript.

## Conflict of Interest Statement

The authors declare that the research was conducted in the absence of any commercial or financial relationships that could be construed as a potential conflict of interest.

## Footnotes

1 http://greenhouse.ucdavis.edu/research/materials/mediafert.html
